# Suitability of Cellulose‐Based Trays With PE/EVOH Coating for Modified Atmosphere Packaging of Salmon, Chicken, and Beef

**DOI:** 10.1111/1750-3841.70505

**Published:** 2025-09-01

**Authors:** Agnete Jordhøy Lindstad, Kloce Dongfang Li, Nusrat Sharmin, Marit Kvalvåg Pettersen

**Affiliations:** ^1^ Department of Food safety and quality Nofima – Norwegian Institute of Food, Fisheries and Aquaculture Research Ås Norway

**Keywords:** cellulose, fiber, food quality, functionality, MAP, packaging, recyclable

## Abstract

Cellulose‐based materials face limitations in fresh meat packaging applications, which require resistance to moist and fatty foods and high environmental humidity, as well as sufficient gas barrier properties for modified atmosphere packaging (MAP). Even with conventional plastic coating, these materials encounter practical challenges such as sealability and structural stability during storage. This study aimed to evaluate the suitability of recyclable cellulose‐based trays coated with polyethylene (PE)/ethylene vinyl alcohol (EVOH)/PE (cellulose/PE/EVOH) for MAP (60% CO_2_/40% N_2_ and 75% O_2_/25% CO_2_) of selected fresh foods of animal sources: salmon, chicken, and beef, against polyethylene terephthalate (PET) trays as control. Headspace gas levels, appearance, and compression strength of packages were assessed during storage, and food quality was analyzed in terms of drip loss, microbiology, and odor. The structure and compression strength of the cellulose‐based trays were greatly affected by the food contact, and the greatest impact was observed during contact with salmon. The compression strength of the cellulose‐based trays was 115 N initially and decreased down to 72 N after contact with food, compared to 95 N for the ones without food contact under similar storage conditions. However, the cellulose‐based trays effectively maintained headspace gas levels comparable to PET trays and did not show negative effects on the quality of the food studied. While combining cellulose‐based trays with PE/EVOH allows for fresh food packaging without compromising food quality, changes in functional material properties during storage may pose challenges to the applicability of such materials throughout the food value chain.

**Practical Applications:**

There is a need for more sustainable food packaging solutions, reducing our abundance of conventional plastics. Hybrid solutions combining cellulose‐based materials with less plastic offer an alternative for challenging food packaging applications in terms of food preservation, but other functionalities must be addressed.

## 1 Introduction

Conventional plastics contribute to global challenges, and a substantial part of these plastics is used by the food packaging sector (Geyer et al. 2017; Rhodes 2019; Yates et al. 2021). Cellulose‐based materials are potential alternatives to plastics for food packaging applications but possess properties like moisture sensitivity and porous structure—that limit their use for most fresh, moist, and greasy products (Andersson 2008; Khwaldia et al. 2010; Vinitskaia et al. 2025). The application of a coating layer can increase the suitability for fresh food packaging applications. However, this can complicate the recycling process and reduce the bio‐based and biodegradable content of the resulting material, depending on the coating (Khwaldia et al. 2010). While there is an increasing interest in cellulose‐based material with biobased and biodegradable coatings, such materials are still in development and currently lack functionalities for fresh food modified atmosphere packaging (MAP) applications (Vinitskaia et al. 2025).

Structural stability and compression strength of whole packages are important for the practical use, transportation, and handling of packages in the food value chain. Poor stackability can cause packaging‐related food loss and waste in distribution and retail (Wohner et al. 2019). Consumers’ willingness to buy packaged meat products can be affected by the appearance of the package (Troy and Kerry 2010). However, the appearance and structural stability of cellulose‐based materials tested for packaging fresh foods are rarely commented on (Vinitskaia et al. 2025).

The effect of relative humidity (RH) on the compression strength of cellulose‐based trays has been studied (Sorensen and Hoffmann 2003). To our knowledge, the impact of food contact on the compression strength of cellulose‐based trays during refrigerated storage has not been previously reported. Interactions between coated cellulose‐based materials and foods during storage can have varying impacts on the mechanical properties of the material, depending on the food product and the coating material (Lindstad et al. 2025). Additionally, mass transfer processes are affected by time (Caner 2011; Dury‐Brun et al. 2007), and the contact time between the material and the packaged food can influence their interactions. Thus, the impact of food contact on material properties may differ between foods of varying expected shelf lives.

Gas barrier properties are among the main functional properties of packaging materials. Traditionally, materials used for fresh meat and fish are typically olefins for effective moisture barrier and sealing properties, combined with ethylene vinyl alcohol (EVOH) for appropriate gas barrier properties (Bauer et al. 2021; Maes et al. 2018). The suitability of materials for MAP applications depends on the gas barrier properties of whole packages. Achieving an appropriate sealing is important, as weak areas in the sealing can lead to gas leakages and food deterioration (Andersson 2008). The gas barrier properties, including sealing, should be sufficiently maintained during storage to keep desired headspace gas levels for the benefits of MAP. Due to the hygroscopic nature of cellulosic materials, humid conditions and moisture sorption may cause structural changes in cellulose‐based packages, including the sealing rim (Niini et al. 2021). It was hypothesized that such changes in cellulose‐based packages during storage could cause defects in the sealing.

Muscle foods (such as meat and fish) release liquid during storage, known as drip loss (mainly consisting of water and proteins), due to changes in their water‐holding capacity (Holck et al. 2014; Huff‐Lonergan and Lonergan 2005). Free liquid in the packages could increase the moisture sorption in the cellulose fiber network during storage and influence the packages’ mechanical strength and structural stability. Hence, it was of interest to investigate to what extent the presence of a liquid absorbent pad could reduce the effect on mechanical properties.

This study aimed to explore the suitability of commercially available cellulose‐based trays with a high barrier (PE/EVOH) coating for packaging and storage of selected fresh muscle foods, using MAP. The emphasis was on preserving food quality and shelf life, in addition to maintaining functional material properties. Salmon, chicken, and beef are products with varying characteristics and shelf life, typically packaged by MAP. The cellulose‐based trays offer an alternative packaging solution with a reduced amount of plastic. The cellulose and PE/EVOH layer can be separated and sorted in their respective streams, allowing both to be mechanically recyclable (European Paper Recycling Council 2022; Pauer et al. 2020). Recyclable PET trays were included as control for comparison to conventional plastic materials. Cellulose‐based packages with and without absorbent pads were included for chicken to test the hypothesis of its impact on the trays’ strength and stability.

## 2 Materials and Methods

### 2.1 Packaging Materials

All food products were packaged in commercially available cellulose‐based (corrugated cardboard) trays coated with a film consisting of PE/EVOH/PE (Jospak Oy, Forssa, Finland). The cellulose‐based trays (cellulose/PE/EVOH) were sealed with Biaxen Eco 65 XX XFP (Wipak Oy, Nastola, Finland), consisting of low‐density PE and EVOH in a multilayer structure (PE/EVOH/PE). Recyclable plastic trays of polyethylene terephthalate (PET) (Faerch, Denmark) were used as reference material in the shelf‐life study, with Mylar 40 OLAF (Petroplast GmbH, Neuss, Germany) as lid film.

The trays were packaged and sealed using a Multivac T200 tray sealer (Multivac, Wolfertschwenden, Germany) with MAP. Food‐grade gases composed of 60% CO_2_ and 40% N_2_, and 75% O_2_ and 25% CO_2_ (Linde Gas AS, Oslo, Norway) were used for MAP. Liquid absorbent pads (Absorber type 109642, MP‐2501 75 × 115 mm black, Faerch, Denmark) were used for all food products and packaging materials, except for one variety of cellulose/PE/EVOH (with chicken) for which no absorbent was used (Table 1).

**TABLE 1:** An overview of the materials, food products, gas compositions, and sampling times used in this study.

Name	Material	Product	Gas	Sampling days
C‐60%CO_2_	Cellulose/PE/EVOH	Salmon	60% CO_2_/40% N_2_	0, 6, 9, 12,16
P‐60%CO_2_	PET	Salmon	60% CO_2_/40% N_2_	0, 6, 9, 12,16
C‐60%CO_2_	Cellulose/PE/EVOH	Chicken	60% CO_2_/40% N_2_	0, 6, 12, 16, 20
C‐60%CO_2_‐w/a^a^	Cellulose/PE/EVOH	Chicken	60% CO_2_/40% N_2_	0, 6, 12, 16, 20
C‐75%O_2_	Cellulose/PE/EVOH	Chicken	75% O_2_/25% CO_2_	0, 6, 12, 16, 20
P‐60%CO_2_	PET	Chicken	60% CO_2_/40% N_2_	0, 6, 12, 16, 20
P‐75%O_2_	PET	Chicken	75% O_2_/25% CO_2_	0, 6, 12, 16, 20
C‐60%CO_2_	Cellulose/PE/EVOH	Beef	60% CO_2_/40% N_2_	0, 12, 20, 28, 35
C‐75%O_2_	Cellulose/PE/EVOH	Beef	75% O_2_/25% CO_2_	0, 12, 20, 28, 35
P‐60%CO_2_	PET	Beef	60% CO_2_/40% N_2_	0, 12, 20, 28, 35
P‐75%O_2_	PET	Beef	75% O_2_/25% CO_2_	0, 12, 20, 28, 35

^a^ w/a: packages without absorbent (all others with).

### 2.2 Food Sample Preparation

Post‐rigor Atlantic salmon fillets (*Salmo salar* L.) were obtained from Salmar (SalMar ASA, Kverva, Norway). The fish was transported and stored in refrigerated conditions before packaging. On the day of packaging (6 days after slaughtering and 2 days after cutting), two randomly selected pieces of fillets with a total average weight of 326.9 ± 9.3 g were packaged in each tray in the gas composition described in Section 2.1.

Chicken breast fillets (pectoralis major) were obtained from Nortura (Nortura SA, Hærland, Norway). After slaughtering, chicken fillets were wrapped in plastic bags and transported in chilled conditions to the research institute, where they were stored at 4°C until packaging. Packaging was performed within 48 h after slaughtering. Fillets were randomly selected from the transport boxes. One and a half fillets with an average total weight of 339.3 ± 8.6 g were packaged in each tray. Samples for further analysis were taken from the whole fillet.

Beef sirloins (aged for 24 days) were obtained from Furuseth (Furuseth AS, Dal, Norway), transported, and stored at refrigerated conditions before packaging. These were cut into pieces with an average weight of 334.7 ± 9.6 g, and one piece of beef was packaged in each tray.

### 2.3 Packaging and Storage

All three products were packaged in cellulose‐based (“cellulose”) and PET trays with modified atmosphere consisting of 60% CO_2_/40% N_2_. Beef and chicken were also packaged in 25% CO_2_/75% O_2_ for both materials, since this gas composition is also commonly used for these two products. A gas volume‐to‐product volume (g/p) ratio of approximately 1:1 and one liquid absorbent pad were used for all combinations. Additionally, chicken samples were packed in cellulose trays without absorbent pad in modified atmosphere of 60% CO_2_/40% N_2_. Sampling days were selected based on the expected shelf life of the different products (Table 1). Packages were stored in refrigerated conditions (4°C and 78 ± 5% RH) for up to 16, 20, and 35 days of storage for salmon, chicken, and beef, respectively.

### 2.4 Analyses

#### 2.4.1 Gas Barrier Properties of Packages

The ambient oxygen ingress rate (AOIR) method (Larsen et al. 2000) was used to assess the oxygen transmission rate (OTR) of the packages under refrigerated conditions with 100% RH inside the packages. The trays were packaged with 100% N_2_ gas and their respective lid films (as described in Section 2.1). The O_2_ concentrations were measured after conditioning and 7–14 days of storage using a CheckMate 3 headspace gas analyzer (PBI Dansensor, Ringsted, Denmark). The OTR values were calculated according to the AOIR method and are expressed as means (*n* = 6).

#### 2.4.2 Headspace Gas Analyses

At each sampling time, the headspace atmosphere of the packages was analyzed in terms of O_2_ and CO_2_ levels (%) using a CheckMate 9900 headspace gas analyzer (PBI Dansensor). Analyzed gas was taken from the packages using a needle through self‐sealing patches. On the day of packaging, eight packages of each variety were analyzed for gas composition immediately after packaging to ensure wanted gas levels. At each sampling time, the headspace atmosphere was measured for each variety (*n* = 3) to identify the gas composition and to exclude packages with gas leakage (before further analyses).

#### 2.4.3 Tray Appearance

**FIGURE 1:**
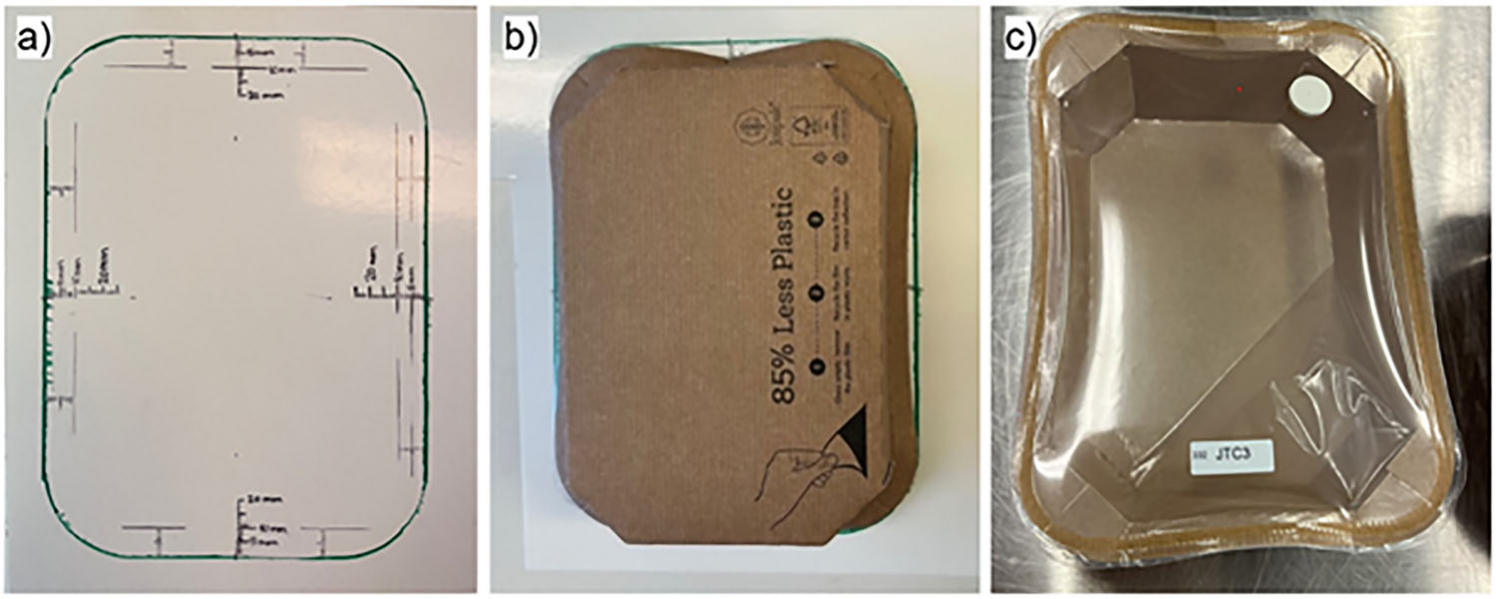
The template used to assess the deformation of the cellulose trays at each sampling time (a), assessment of the deformation of a cellulose tray (b), and an empty cellulose tray with visible delamination of coating from the cellulose (“air pocket”) (c).

Different materials (e.g., cellulose and plastics) have different properties that can influence their functionality as food packaging. The appearance of the cellulose‐based trays was evaluated at each sampling time. Deformation of the cellulose trays was assessed by measuring the deviation from the initial shape. A template with the original outer perimeter was created (Figure 1a). The samples were placed upside down onto the template to measure the distance (mm) from the original outer circumference of the tray to the point with the largest deformation of each side, for all four sides of the trays (Figure 1b). The deformation was calculated as the sum of all four sides of the tray, given in millimeters. The results are presented as mean values with standard deviation (*n* = 3). Additionally, the number of trays showing delamination of coating from the cellulose fiber part, resulting in “air pockets,” was assessed (shown for an empty tray in Figure 1c).

**FIGURE 2:**
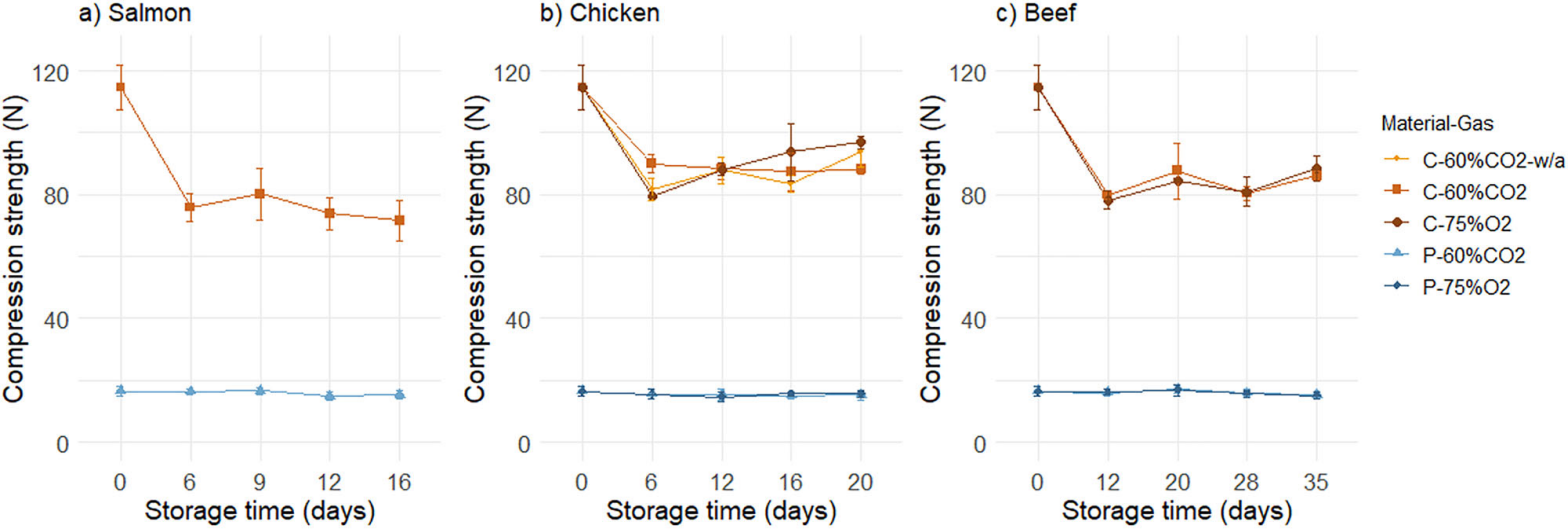
The compression strength of cellulose (“C”) and PET (“P”) trays before (Day 0) and after 6–35 days of storage under 4°C/78% RH with salmon (a), chicken (b), and beef (c), with different gas compositions (60% CO_2_/40% N_2_ [“60%CO2”] and 75% O_2_/25% CO_2_ [“75%O2”]). C‐60%CO2 trays with chicken were packaged without absorbent (“w/a”), all others with absorbent. Values are presented as means (*n* = 3), with standard deviation represented by the error bars.

#### 2.4.4 Compression Strength of Trays

A compression test on empty trays was conducted using a modified method of the TAPPI 804 standard. An Instron Universal Testing Machine Model 5944 (Instron, US) with a load cell of 2 kN was equipped with a circular metal plate of diameter 5.7 cm. Trays were placed upside down on a metal base in a set position. The circular plate was moving perpendicularly down onto the middle part of the tray bottom at a rate of 100 mm/min, while load (N) and deformation (mm) were recorded. Tray deformation (reduction in height) of 10 mm was considered the critical deformation, and the highest load until this point was regarded as the tray's compression strength. Initial compression strength was determined before the experiment for each material (*n* = 5). The compression strength for each sampling time (after removing the product) is expressed as the mean with standard deviations (*n* = 3).

#### 2.4.5 Drip Loss

The initial product weight in each package was determined on the day of packaging. At each sampling time, the weight of excessive liquid (drip loss) in the package was determined by weighing the package before and after removing excessive liquid using tissue paper. For packages with absorbent, the drip loss in the absorbent was included in the calculation. Results are given as the percentage (%) of initial product weight, presented as means with standard deviation (*n* = 3).

#### 2.4.6 pH, Water Activity, and Dry Matter

pH was measured at each sampling time after conditioning at room temperature, using a Beckman 31 pH meter with a Xerolyte electrode (Mettler‐Toledo AG, Grefensee, Switzerland). The water activity of food samples was assessed on the day of packaging (Day 0). Circular samples of diameter 3.5 cm were cut and measured in an Aqualab 3 Water Activity Meter (Aqualab by Addium Inc., Pullman, WA, USA). The dry matter content of the food samples was determined at each sampling time by macerating the food and oven drying approximately 6 g of each sample on Petri dishes at 105°C for 18 h. The weight loss during drying corresponds to the water content of the samples. The initial weight and the water content were used to calculate the dry matter content, expressed as percentage (%) of the initial weight. Results are presented as the mean value with standard deviation (*n* = 3).

#### 2.4.7 Microbiological Analyses

For assessment of microbiological quality of the food samples, analyses of typical spoilage bacteria (Chan et al. 2024; Stanborough et al. 2017; Säde et al. 2013) were conducted on the day of packaging (*n* = 5) and at each sampling time (*n* = 3). Sterile scalpels were utilized to obtain samples of 3 × 3 × 1 cm (including surface). The samples were weighed, macerated, and diluted in peptone water at a ratio of 1:10.

For chicken and beef, the diluted samples were spread on the following media (by the Nordic‐Baltic Committee on Food Analysis [NMKL] standardized methods) with a spreader or using Whitley Automated Spiral Plater (WASP) (Don Whitley Scientific Ltd., West Yorkshire, UK):

‐ PCA Oxoid CM 0463 (plate count agar; Difco, Difco Laboratories, Detroit, MI, USA) and incubated anaerobically at 20°C for 72 h, for total viable count (TVC).
‐ Streptomycin thallous acetate actidione agar base (STAA; CM 0881 with selective supplement SR 0151E; Oxoid, Hampshire, UK) and incubated at 25°C for 48 h, for *Brochothrix* sp.
‐ Violet red bile glucose agar (VRBGA; CM 1082, Oxoid) incubated at 37°C for 24–48 h, for *Enterobacteriaceae*.

For salmon, diluted samples were spread on iron agar (Lyngby; CM 0964, Oxoid) with additional NaCl to a total of 1% to promote the growth of marine bacteria. Samples were incubated aerobically at 15°C for 5–7 days, and the total number (TVC) and the number of black colonies (hydrogen sulfide‐producing bacteria) were counted.

The microbial levels were expressed as log cfu/g. The detection limits were 10^2^ cfu/g for all analyses, except for *Enterobacteriaceae*, for which it was 10^1^ cfu/g.

For the two last sampling points of each product, the microbial composition was analyzed with matrix‐assisted laser desorption ionization time‐of‐flight mass spectrometry (MALDI‐TOF MS) for the relative distribution of different bacterial genera. For each sample, 10 colonies from the PCA plates for chicken and beef, and iron agar plates for salmon, were randomly selected. Mass spectra was obtained by the common direct transfer protocol. Approximately 0.1 mg of cell material was transferred directly from each colony to a target plate and overlaid with 1 µL of matrix solution (10 mg/mL a‐cyano‐4‐hydroxycinnamic acid in 50% acetonitrile and 2.5% trifluoroacetic acid). A Biotyper MALDI‐TOF mass spectrometer (Bruker Daltonics, Bremen, Germany) was used for the MS analysis, with MBT Compass 4.1 and FlexControl 3.4 software (Bruker Daltonics). The instrument was calibrated with the Bacterial Test Standard (Bruker Daltonics). The FlexControl software was used for automatic measurements of all MS spectra, following the standard measurement method for microbial identification, MBT‐autoX.axe autoExecute. Classification was conducted using the BioTyper 3.1 software (Bruker Daltonics) with 10833 MSPs Library (released January 2022). Score values were interpreted as follows: <1.7 as unreliable identification, 1.7–2.0 as reliable genus identification, and >2.0 as secure genus and reliable species identification. Results are presented for each sample at genus level (only score values >1.7).

#### 2.4.8 Odor Assessment

An assessment of odor of raw food was conducted for all three products stored in both gas compositions with absorbent at each sampling point. Odor samples (including surface) were cut from the same section of the fillets for each variety throughout the experiment. Samples were cut from the back (top part) of the salmon fillets and the “tale” of the chicken fillets. At each sampling time, the panel was provided with a reference sample (frozen on the day of packaging and thawed within 24 h before the odor assessment). This was used as a reference for the odor of a fresh product and was not given any score. A similar sample was included in the assessment as a control sample (served randomized). The samples were conditioned at room temperature, given three‐digit codes, kept under metal lids, and served randomized. A semitrained panel of minimum five people rated the samples from 5 to 1, where 5 was the best score (for fresh odor), and 1 was the worst, meaning that the sample was regarded unacceptable for consumption. A score of 3 was defined as the acceptability threshold, where the food sample was noticeably altered compared to fresh, but still acceptable for consumption after heat treatment. The study was reviewed by Nofimas Ethical board, and informed consent was obtained from each person before they participated in the study. The mean value of the scores for each sample (rated by five to seven people) was used to calculate the mean value with standard deviation (*n* = 3).

### 2.5 Statistical Analysis

R software (R 4.2.2, R Foundation for Statistical Computing, Vienna, Austria) was used for statistical analysis. For each food product, an overall ANOVA on each response variable was performed using the aov() function in R. The overall ANOVA for the different samples was conducted as follows: For chicken and beef, a three‐way ANOVA with material, gas composition, and time was used to identify the main effects and their interactions. For salmon, a two‐way ANOVA with material and time was used. Additionally, a two‐way ANOVA was used to identify the effect of absorbent, time, and their interaction on response variables for chicken in C‐60%CO_2_.

One‐way ANOVA with post hoc comparisons was conducted using Tukey's honest significant difference (HSD) to generate compact letter displays (CLDs) indicating significant differences between factor levels. Each combination of material, gas composition, and sampling time was regarded as a separate level. The presence of absorbent in C‐60%CO_2_ was analyzed separately, with the presence/absence of absorbent at each sampling time as separate levels.

In addition, material properties after storage were compared between products in 60% CO_2_ (which was used for all three products), using a three‐way ANOVA with product, material, and time as factors, to identify the main effects and their interactions on deformation (cellulose‐based trays) and compression strength. A level of significance of 0.05 was used for all tests.

## 3 Results and Discussion

### 3.1 Material Properties

#### 3.1.1 Gas Barrier Properties of Packages

The OTR of the cellulose/PE/EVOH and PET packages at 4°C/78% RH were 0.1 and 0.4 mL O_2_/package/day, respectively. The OTR of the cellulose‐based trays was significantly lower compared to PET, as expected due to the PE/EVOH layer (Maes et al. 2018).

#### 3.1.2 Compression Strength

The initial compression strength of cellulose trays was 115 N, while that of PET trays was 16 N. This was likely related to their varying thickness; the cellulose/PE/EVOH trays were approximately 850 µm thick, and the PET trays approximately 300 µm. The high compression strength of the cellulose trays was expected (Leminen et al. 2020). After 20 and 35 days of storage, the compression strength of cellulose trays without product was reduced by 13% and 17%, respectively (results not shown). The compression strength of cellulose‐based trays is known to decrease under exposure to high RH, due to the hygroscopic nature of cellulose fibers and softening effect of moisture in the fiber network (Alava and Niskanen 2006). Sorensen and Hoffmann (2003) reported that the compression strength of cellulose trays conditioned at 97% RH was less than one third of that at 33% RH. Thus, a decrease in compression strength of cellulose trays during storage at 78% RH was expected. Moreover, the compression strength of cellulose trays with food contact decreased up to 37% (Figure 2), significantly more compared to 17% for those without product stored under the same conditions. These results suggest that food contact has a great impact on the compression strength of the cellulose trays, despite the use of conventional PE/EVOH as a barrier between the cellulose and food.

**FIGURE 3:**
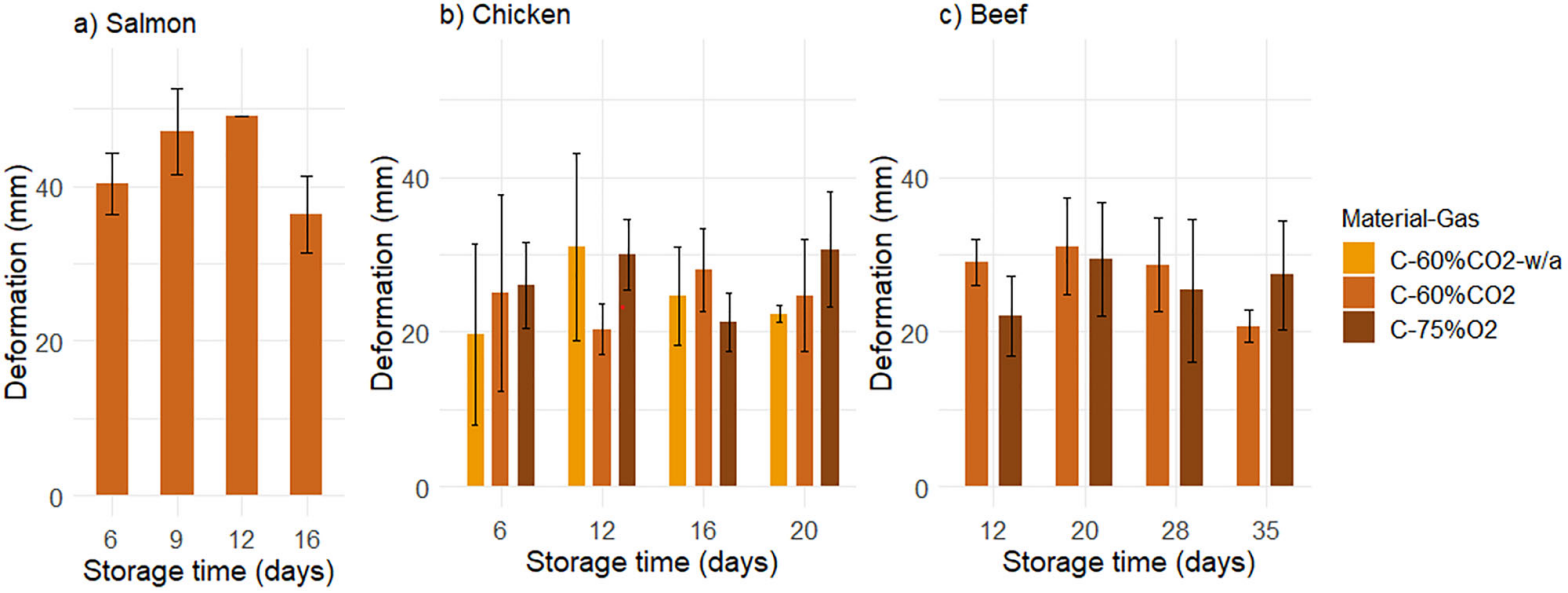
The deformation of cellulose tray walls as the sum of all four sides (mm) prior to opening the package, after 6–35 days of storage under 4°C/78% RH for salmon (a), chicken (b), and beef (c). Chicken and beef were packaged in 60% CO_2_/40% N_2_ (“60%CO2”) and 75% O_2_/25% CO_2_ (“75%O2”). “w/a” indicates packages without absorbent. Values are presented as means (*n* = 3), with standard deviation represented by the error bars.

Furthermore, two‐way ANOVA of C‐60%CO_2_ trays for all three products during storage showed a significant effect of the product. While the compression strength of C‐60%CO_2_ with chicken and beef decreased by approximately 15%–30%, a decrease of 30%–40% was observed with salmon. (Selected curves of compression strength are presented in the Supporting Information.) Possibly, the greater impact of salmon is linked to its higher fat content and different fat composition compared to chicken and beef. Fat can interact with hydrophobic polymers like PE (Arvanitoyannis and Bosnea 2004), and oils can penetrate through PE coating on paperboard (Mikriukova et al. 2022), while EVOH is sensitive to moisture (Mokwena et al. 2011). Usually, concerns about moisture from moist foods interacting with the cellulose fiber network are reported (Rhim 2010; Siracusa 2012; Sorensen and Hoffmann 2003). In the present study with PE/EVOH combined with cellulose, the results indicate that moist products with substantial amounts of fat (in this case, salmon) can have even greater effects on the tray strength than food products of higher moisture content and less fat (in this case, chicken and beef) (Section 3.2.3).

For C‐60%CO_2_ with chicken, with and without absorbent (C‐60%CO_2_‐w/a), it was hypothesized that the free liquid in the trays without absorbent could lead to increased moisture sorption (in the cellulose fiber network) and thereby reduce the compression strength. However, there was not a main effect of absorbent on the compression strength. There was a significant interaction between absorbent and time. The packages without absorbent initially showed a greater strength reduction compared to 20 days of storage.

The compression strength of trays was not affected by gas composition. Overall ANOVA showed a significant effect of material, time, and their interaction, for all products. The strength of cellulose trays mainly decreased during the initial days of storage, while PET remained relatively stable. This suggests that the type of product (and environmental conditions, i.e., RH) is more important than the storage time and gas composition used for MAP. Still, the compression strength of cellulose trays remained higher than that of PET.

#### 3.1.3 Tray Appearance

As moisture has a softening effect on cellulosic fiber networks (Alava and Niskanen 2006), the high RH during storage may have influenced the structure of the cellulose trays, including the deformation of tray walls. This was supported by the deformation of empty trays (packaged with 60% CO_2_), which was 6–18 mm during 35 days of refrigerated storage. However, trays with all three products showed greater deformation compared to empty trays during storage (Figure 3). This indicates that the shape of the cellulose trays was greatly affected by the product during storage, in addition to the surrounding RH. The deformation of trays with salmon reached 49 mm after 12 days, followed by a decrease. The deformation of trays with chicken and beef was approximately 20–30 mm, respectively, throughout their storage times. The greater deformation with salmon compared to chicken and beef, likely related to its higher fat content, is in line with the changes in compression strength discussed above. Niini et al. (2021) observed similar deformation of PET‐coated cellulose‐based trays with oatmeal.

The tray deformation during storage may partly be due to the formation of underpressure and following reductions in headspace volume for packages with muscle products using MAP with CO_2_. Substantial amounts of CO_2_ are dissolved into all three products at the beginning of the storage period. This phenomenon has previously been reported to lead to packaging collapse and “squeezed” packages (for plastic trays) (Fernández et al. 2010; Holck et al. 2014). There was no difference in tray deformation between gas compositions. Furthermore, oxygen is consumed in meat products (McMillin 2008), contributing to similar results for both gas compositions.

**FIGURE 4:**
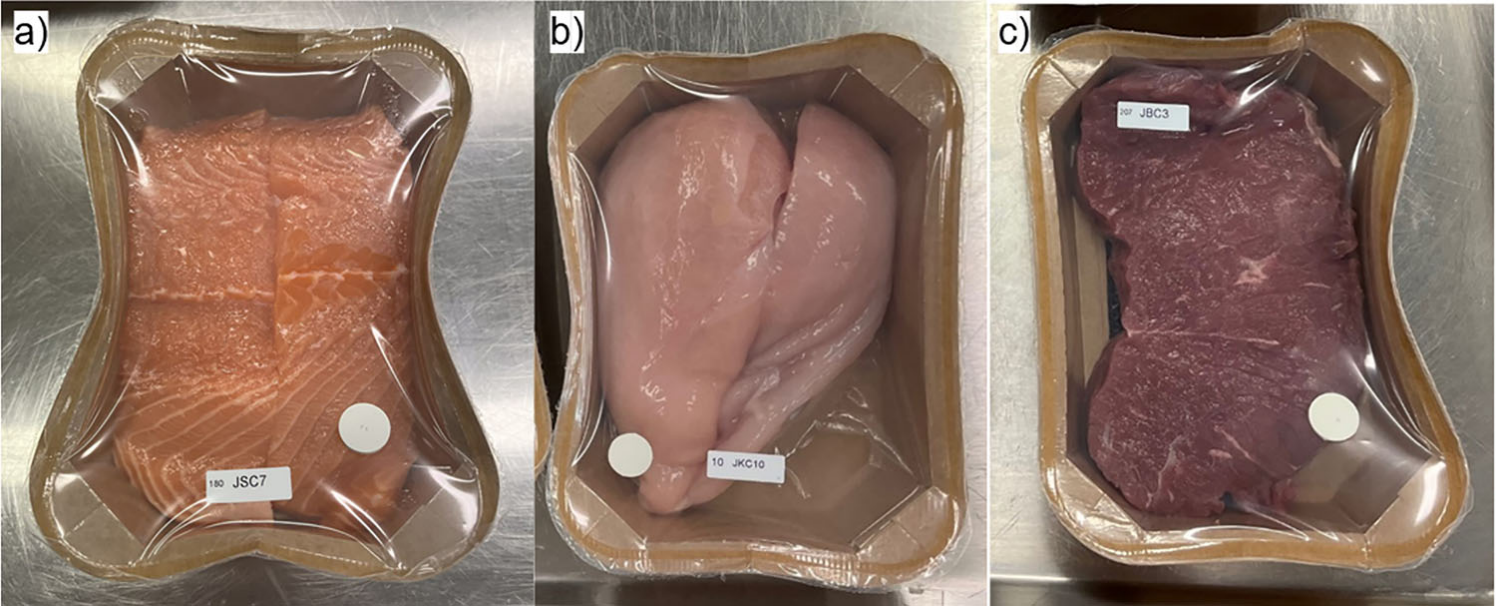
Cellulose trays with salmon (a), chicken (b), and beef (c) in 60% CO_2_/40% N_2_ after 9–12 days of storage at 4°C/78% RH, showing deformation of tray walls and “air pockets” (bottom right corner of trays with chicken and beef).

Partial delamination of the coating from the cellulose part of the trays, forming “air pockets,” was observed for all products during storage (Figure 4). These were likely formed due to the underpressure in the packages in combination with moisture sorption in the cellulose, leading to a reduction in headspace volume. Usually, the lid film is the most flexible part of a semirigid tray, and the volume reduction can result in “suction” of the lid film down onto the packaged product (Rotabakk et al. 2006), but for the cellulose trays, the PE/EVOH coating was also flexible and adhesion to the cellulose part was relatively weak.

Furthermore, the overall ANOVA of tray deformation with the different products in C‐60%CO_2_ showed that the type of product had a significant effect. Despite the shorter storage time of salmon compared to beef, the deformation was greater for salmon. These differences are likely connected to the interactions between product and packaging, as previously mentioned. While none of the salmon packages showed air pockets until the last sampling time, all chicken and beef packages did from day 12 and 20, respectively (results not shown). This can explain the change in the deformation of salmon packages, which decreased after 16 days of storage, as the number of trays with air pockets increased simultaneously. The salmon fillets covered more of the tray bottom, and thus, there may have been more resistance to the formation of air pockets, possibly increasing the impact of underpressure on deformation.

Tray deformation may pose challenges in different ways. Packaging shape or geometry can influence stacking strength (Fadiji et al. 2016; Lu et al. 2023), and reduced stacking strength can lead to food waste (Wohner et al. 2019). Results from both the compression strength and deformation of trays during storage suggest that the cellulose trays may be more suitable for chicken and beef compared to salmon. The deformation of tray walls and air pockets in cellulose trays can make consumers skeptical, questioning the food safety and suitability of this packaging solution.

### 3.2 Food Quality

#### 3.2.1 Headspace Gas

**FIGURE 5:**
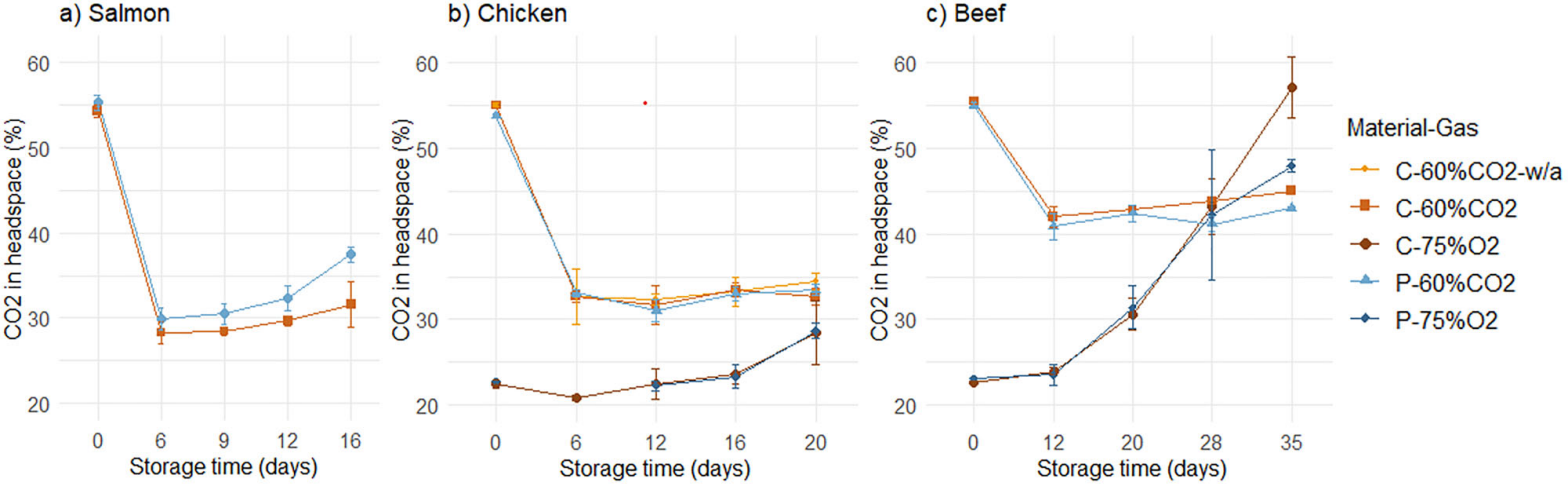
CO_2_ levels (%) in the headspace of salmon (a), chicken (b), and beef (c) packages on the day of packaging and after 6–35 days of storage under 4°C/78% RH, in cellulose (“C”) and PET (“P”) trays with 60% CO_2_/40% N_2_ (“60%CO2”) and 75% O_2_/25% CO_2_ (“75%O2”) gas. “w/a” indicates packages without absorbent. Values are presented as means (*n* = 3), with standard deviation represented by the error bars.

The CO_2_ content in the headspace (%) of C‐60%CO_2_ and P‐60%CO_2_ packages initially decreased for all products (Figure 5). This was likely due to CO_2_ dissolving in the meat (Chan et al. 2024; Pettersen et al. 2011; Sivertsvik et al. 2003). Different levels of CO_2_ were observed for the products, with the lowest level in salmon (30%) and chicken (30‐35%) and the highest level in beef (40%–45%). For the CO_2_ content in salmon packages, there was a significant interaction between material and time. After 16 days of storage, the CO_2_ concentration in P‐60%CO_2_ was higher compared to C‐60%CO_2_. For chicken and beef, there was no effect of material on the CO_2_ and O_2_ levels in 60%CO_2_ packages.

For the 75%O_2_ packages with chicken and beef, there was an increase in CO_2_ and a decrease in O_2_ content in the headspace during storage. This was likely due to oxygen consumption (oxidation reactions in meat and by microorganisms present on the meat surface) leading to a higher relative amount of CO_2_ (Murphy et al. 2013; X. Y. Yang et al. 2016). There was not a main effect of material on the gas levels. However, at the last sampling time for beef, the O_2_ level in C‐75%O_2_ was significantly lower compared to P‐75%O_2_, while the opposite was observed for the CO_2_ level. Storage under refrigerated conditions resulted in changes in material properties of cellulose/PE/EVOH, especially with product (Section 3.1.3), and may also have influenced the gas barrier properties. Similar gas levels in cellulose and PET trays were expected due to the similar magnitude of their OTR (although significantly different).

#### 3.2.2 Drip Loss

**FIGURE 6:**
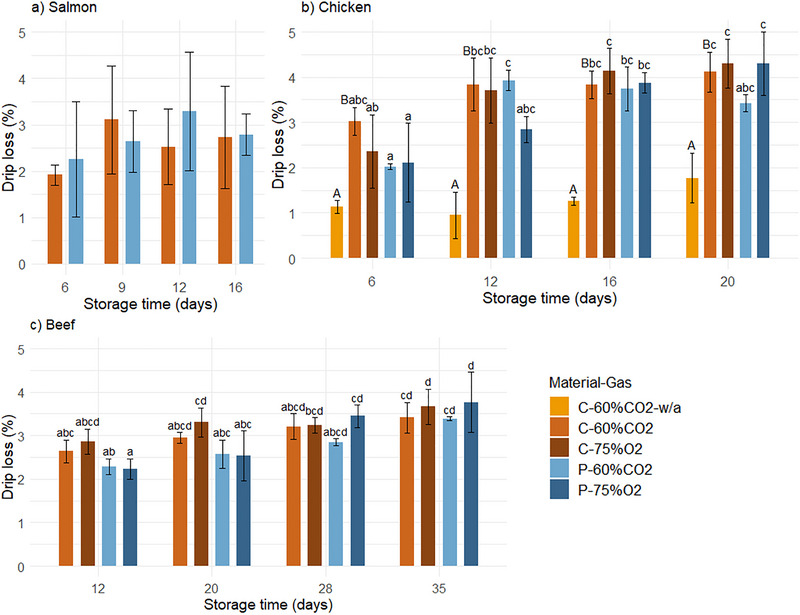
The drip loss (%) of salmon (a), chicken (b), and beef (c) packaged in cellulose/PE/EVOH (“C”) and PET (“P”) trays with 60% CO_2_/40% N_2_ (“60%CO2”) and 75% O_2_/25% CO_2_ (“75%O2”) gas during storage under 4°C/78% RH. Values are given as means (*n* = 3), with standard deviation represented by the error bars. Significant differences between samples are indicated by letters (those sharing the same letter are not significantly different at *p* < 0.05). Significant differences between C‐60%CO2 with and without absorbent (“w/a”) for chicken are indicated by capital letters.

Drip loss is excessive moisture that is naturally released from the muscle during storage. Although unavoidable, it should be minimized due to the potential negative impact on consumer perception (Droval et al. 2012). The drip loss of all products increased during storage (Figure 6), as expected (Hansen et al. 2009; Sivertsvik et al. 2003). As previously mentioned (Section 3.1.3), the dissolution of CO_2_ in muscle products can result in a reduced headspace volume, which can cause physical pressure on the packaged product and increase its drip loss (Holck et al. 2014; Payne et al. 1998; Rotabakk et al. 2006). In addition, CO_2_ can permeate through the material and increase this effect, depending on the gas barrier properties and concentration difference between the headspace and the surroundings (Siracusa 2012). The overall ANOVA showed a main effect of material on the drip loss of chicken and beef, as well as an interaction between material and time for beef. However, the differences in drip loss in cellulose and PET (with the same gas composition) were not significantly different at any sampling time.

The gas composition had a main effect on the drip loss of beef, as previously reported for other gas compositions (Ercolini et al. 2006). There was also a significant interaction between gas composition and time for the drip loss of chicken. While the drip loss of chicken in 75% O_2_ was significantly higher after 20 days of storage compared to 6 days, it was not for chicken in 60% CO_2_. Pettersen et al. (2021) reported that the drip loss of chicken breast fillets packaged in 75% O_2_ gas was lower compared to 60% CO_2_ (but not significantly different at any sampling time). The effects of gas composition and material can be related to the headspace volume reduction of the packages during storage.

Other studies have shown that the collapse or squeeze of packages can increase the drip loss of salmon (Chan et al. 2024; Fernández et al. 2010). In the current study, there was a substantial deformation of C‐60%CO_2_ trays with salmon (greatest among the tested food products) (Section 3.1.3). However, the material did not significantly affect the drip loss of salmon, indicating that the deformation in C‐60%CO_2_ did not result in additional physical pressure on the salmon fillets compared to P‐60%CO_2_.

Furthermore, the drip loss of chicken in C‐60%CO_2_‐w/a (without absorbent) was significantly lower compared to C‐60%CO_2_ with absorbent, throughout the storage time. This could be expected, as increasing the number of absorbents has been shown to lead to increased drip loss of chicken because absorbents draw excess liquid from the product (Pettersen et al. 2021).

#### 3.2.3 pH, *a*_w_, and Dry Matter

The pH of all products decreased during storage but was not affected by material. Dissolved CO_2_ can have an acidic effect, reducing the pH of the muscle products. The gas compositions used in our study (60% CO_2_ vs. 25% CO_2_) may have influenced the amount of CO_2_ dissolved in the meat fillets (Rotabakk et al. 2010) but did not result in different pH values, in accordance with literature (Rotabakk et al. 2006; X. Y. Yang et al. 2016).

On the day of packaging, the *a*_w_ of salmon was 0.98, that of chicken was 0.99, and that of beef was 0.98. The dry matter of salmon decreased from 41.3% on the day of packaging to 34.0% in PET and 36.9% in cellulose after 16 days of storage. On the other hand, the dry matter of chicken increased, as previously reported (Pettersen et al. 2021), from 23.1% on the day of packaging to 23.3%–24.4% after 20 days. For beef, the dry matter changed from 26.2% to 25.1%–26.6% during storage.

There was no significant effect of material, gas composition, or the presence of absorbent. This suggests that the interactions between the packaged products and cellulose trays indicated by the changes in functional properties (Section 3.1.2 and 3.1.3) did not influence pH, *a*_w_, or dry matter.

#### 3.2.4 Microbiological Quality

As expected, the TVC of all products increased with time but was not significantly affected by the material or gas composition. A TVC of 6–7 log cfu/g is commonly used as a threshold for microbial spoilage of meat and fish products (Chan et al. 2021; Fernández et al. 2010). However, the microbial spoilage potential depends on the types of bacteria present, not only the amount. MAP with CO_2_ can inhibit the growth of common spoilage bacteria on muscle products and usually leads to the dominance of lactic acid bacteria that are resistant to CO_2_ (Hansen et al. 2023; Rotabakk et al. 2006; Sarfraz et al. 2021). The CO_2_ concentration in the headspace does not necessarily affect the TVC, but the type of bacteria that will grow.

**FIGURE 7:**
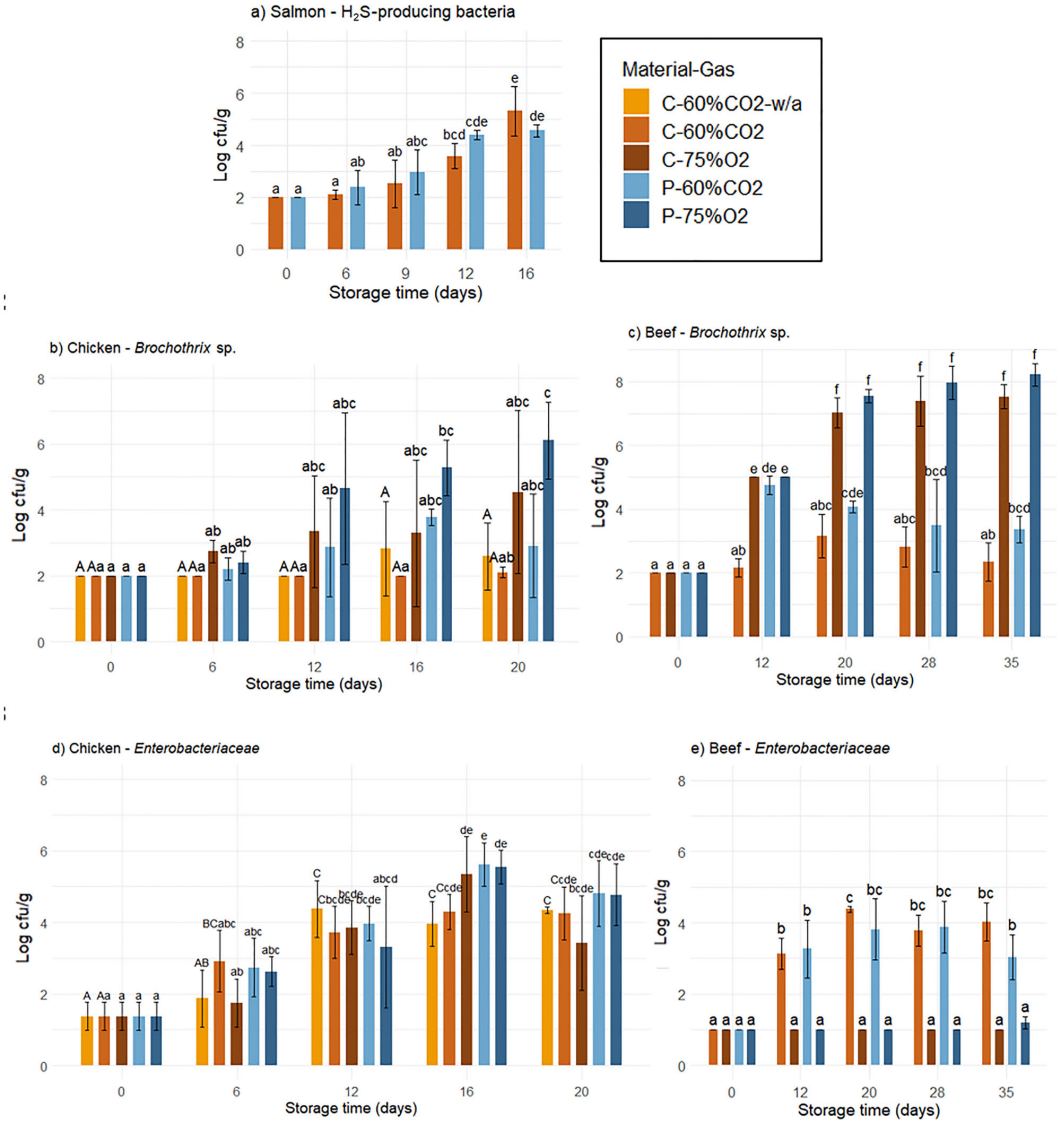
The H_2_S‐producing bacteria in salmon (a), *Brochothrix* sp. in chicken (b) and beef (c), and *Enterobacteriaceae* in chicken (d) and beef (e) on the day of packaging and after 6–35 days of storage under 4°C/78% RH, packaged in cellulose/PE/EVOH (“C”) and PET (“P”) trays with 60% CO_2_/40% N_2_ (“60%CO2”) and 75% O_2_/25% CO_2_ (“75%O2”). Values are given as means (*n* = 3), with standard deviation represented by the error bars. Significant differences between samples are indicated by letters (those sharing the same letter are not significantly different with *p* < 0.05). Significant differences between C‐60%CO2 with and without absorbent (“w/a”) for chicken are indicated by capital letters. For (c), on Day 12, the *Brochothrix* sp. plates for 75% O_2_ were overgrown (not sufficiently diluted). Levels are set at 5 log cfu/g based on the dilution.

For salmon, the TVC was 2.4 log cfu/g on the day of packaging and gradually increased to 7.7 log cfu/g after 16 days of storage in both materials. H_2_S‐producing bacteria are typical spoilage bacteria on salmon, including bacteria like S*hewanella* sp. (Sivertsvik et al. 2003), and the H_2_S they produce contributes to the characteristic odor of rotting fish (Yan et al. 2024). For CO_2_‐sensitive bacteria like *Shewanella* sp., the use of MAP with CO_2_ can both inhibit microbial growth and reduce H_2_S production. Due to the similar barrier properties and headspace gas levels of the different packaging materials in our study, comparable levels of H_2_S‐producing bacteria were expected (Figure 7).

**FIGURE 8:**
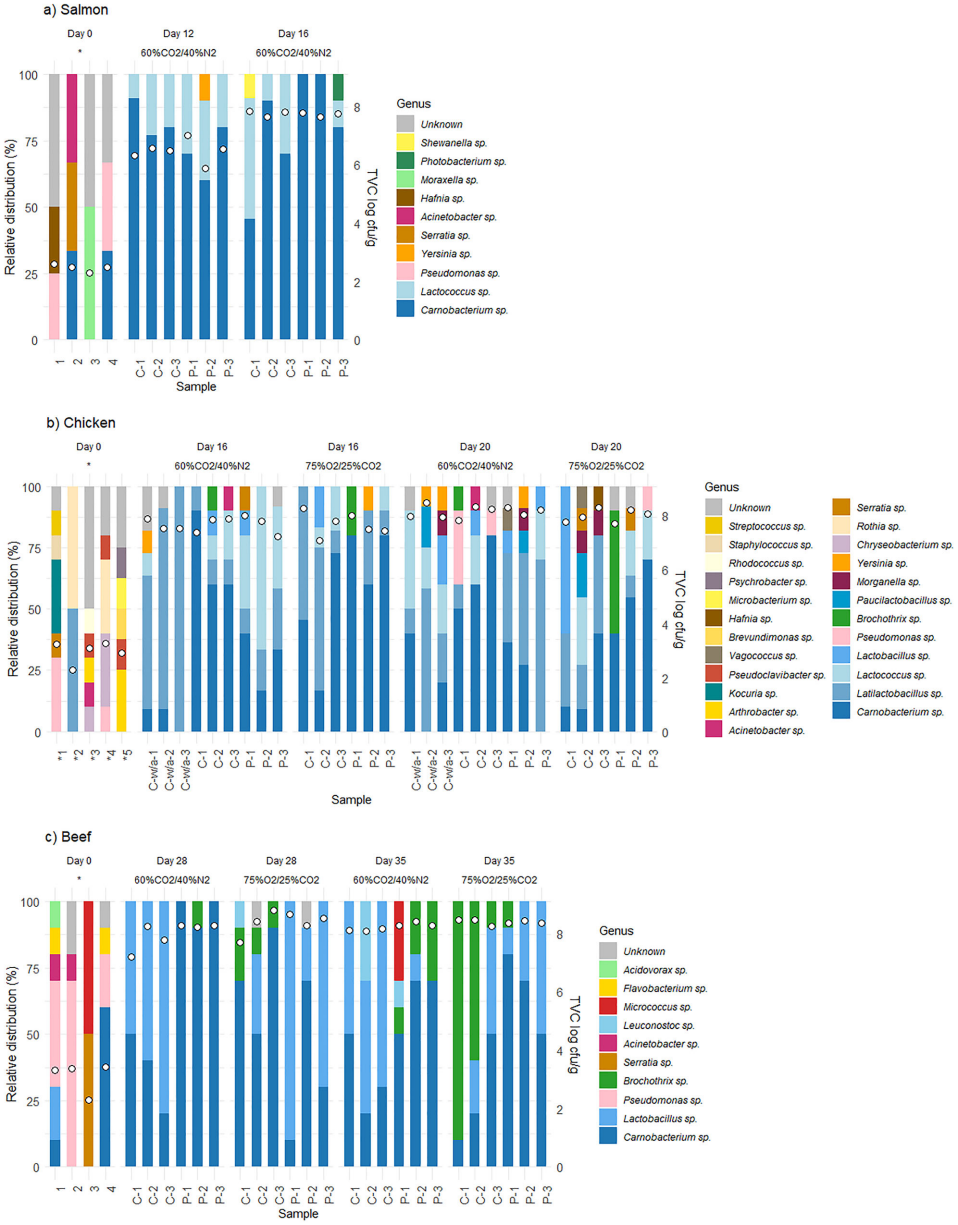
Relative distribution of bacterial genera on salmon (a), chicken (b), and beef (c) on the day of packaging (Day 0) and after 12–35 days of storage under 4°C/78% RH in cellulose/PE/EVOH (“C”) and PET (“P”) trays with 60% CO_2_/40% N_2_ and 75% O_2_/25% CO_2_ gas. “w/a” indicates packages without absorbent. Each bar represents one sample. The total viable count (TVC) of each sample is marked with a circle.

For chicken and beef, the TVC was 3.0 and 2.7 log cfu/g on the day of packaging, respectively. The TVC of chicken increased to approximately 5 log cfu/g after 6 days of storage and reached a relatively high level (7.1–7.6 log cfu/g) after 12 days of storage, similar to beef (6.8–7.3 log cfu/g), followed by an increase to approximately 8 log cfu/g until the end of storage (Figure 8). There were no significant differences between the packaging solutions at each sampling time for chicken according to one‐way ANOVA. For beef, there was a significant interaction between gas and time, with higher TVC in C‐75%CO_2_ compared to C‐60%CO_2_ after 12 days, while the opposite was observed after 35 days of storage. However, the differences can be regarded as marginal. The formation of off‐odors causing spoilage of raw meat is known to be dominated by certain bacterial genera, depending on the gas composition (Hansen et al. 2023). Selective media were used to analyze the levels of spoilage bacteria during storage.

The levels of *Brochothrix* sp. in chicken and beef increased with time, from below the detection limit (2.0 log cfu/g) on the day of packaging (Figure 7). Overall ANOVA showed a main effect of the material, gas composition, and the interaction between gas composition and time. For chicken, there was also a significant interaction between material and time. The effect of gas composition was expected, as the growth of *Brochothrix* sp. can be inhibited or delayed by CO_2_ in the packaging atmosphere (Holck et al. 2014; Pettersen et al. 2021; Pin et al. 2002; Sarfraz et al. 2021). Moreover, CO_2_ inhibition can extend the lag phase of microbial growth, as supported by the interaction between gas composition and time. The levels of *Brochothrix* sp. were lower in 60% CO_2_ compared to 75% O_2_, and the effect was more pronounced for beef compared to chicken. While the level in chicken reached 6.1 log cfu/g in P‐75%O_2_, that in beef reached 8.3 log cfu/g in P‐75%O_2_. The main effect of material may have resulted from the different gas barrier properties (Section 3.1.1), but the differences were not significant at any sampling time.

The levels of *Enterobacteriaceae* in chicken increased (from 1.4 log cfu/g) to approximately 4–5.5 log cfu/g during storage for all packaging solutions and were not significantly affected by material or gas composition. Similar results were observed by Pettersen et al. (2021), who reported no significant effect of gas composition (studying the same gas compositions of 60% CO_2_/40% N_2_ and 75% O_2_/25% CO_2_) on the growth of *Enterobacteriaceae* on chicken, while for CO_2_/N_2_ compositions, it has been reported to increase with decreasing amount of CO_2_ (Holck et al. 2014). After 16 days of storage, the *Enterobacteriaceae* levels in P‐60%CO_2_ were >1 log higher compared to C‐60%CO_2_, but not significantly different. For chicken in general, the counts on selective media (*Brochothrix* sp. and *Enterobacteriaceae*) did not add up to the TVC, indicating that there were other bacteria present at relatively high levels, such as lactic acid bacteria (Pettersen et al. 2021).

On the other hand, the growth of *Enterobacteriaceae* in beef was significantly affected by gas composition, with higher levels in 60% CO_2_ (3–4 log cfu/g) compared to 75% O_2_ (below detection limit). These results may indicate that high oxygen levels can delay the growth of *Enterobacteriaceae*. This is in accordance with some literature (Ercolini et al. 2006), but not with other (Esmer et al. 2011; X. Y. Yang et al. 2016), and not with the findings for chicken in this study. Furthermore, the growth of different bacteria can depend on the meat product, providing different conditions and nutrients for microbial growth. The high levels of *Brochothrix* sp. on beef in 75% O_2_ were similar to the TVC of the same samples. The dominance of some bacteria may inhibit the growth of others, in this case, *Enterobacteriaceae*, due to lacking nutrients on the meat surface.

The microbiological quality of chicken in C‐60%CO_2_‐w/a (without absorbent) was not significantly different from chicken packaged in C‐60%CO_2_ with absorbent. A similar pattern was observed by Pettersen et al. (2021), reporting that the number of absorbents did not affect the microbiological quality of chicken during storage, disproving a general perception that free liquid inside packages reduces microbiological quality.

The relative distribution of bacteria on all three products changed during storage (Figure 8). Over time, the storage conditions favored lactic acid bacteria, likely due to their tolerance to CO_2_ and their ability to grow under anaerobic, refrigerated conditions (Chan et al. 2024). In particular, *Carnobacterium* sp. was frequently identified in all three products, together with other lactic acid bacteria (*Lactococcus* sp. in salmon, *Latilactobacillus* sp. and *Lactococcus* sp. in chicken, and *Lactobacillus* sp. in beef). *Carnobacterium* sp. are usually among the dominating bacteria during refrigerated storage of modified‐atmosphere‐packaged salmon (Chan et al. 2024; Leisner et al. 2007), chicken (Leisner et al. 2007), and beef (Ercolini et al. 2009).

For salmon, common H_2_S‐producing bacteria (*Yersinia* sp., *Photobacterium* sp., and *Shewanella* sp.) were occasionally identified, without any systematic trend with material. Due to its resistance to CO_2_, *Photobacterium* sp. is usually among the dominating genera on salmon in 60% CO_2_/40% N_2_ (Chan et al. 2024), but was only detected in one sample in the current study.

The relative distribution of bacteria on chicken showed greater diversity compared to the other products. After 20 days of storage of chicken, lactic acid bacteria still dominated, but various potential spoilage bacteria were also identified. Several genera belonging to *Enterobaceriaceae* were found in both gas compositions: *Morganella* and *Yersinia* sp. in 60% CO_2_, and *Morganella*, *Serratia*, and *Hafnia* sp. in 75% O_2_, as expected (Holck et al. 2014; Höll et al. 2016; Säde et al. 2013). Additionally, *Brochothrix* sp. and *Pseudomonas* sp. were identified. These findings were in accordance with the plate counts of the selective media.

For beef, the relative amount of *Brochothrix* sp. seemed to increase with time, in particular for C‐75%O_2_ (after 35 days), similar to what was reported for beef packaged with 50% O_2_ (J. Yang et al. 2022). However, the plate counts of *Brochothrix* sp. indicated dominance in all 75%O_2_ samples, while the relative distribution showed high amounts of lactic acid bacteria, especially for P‐75%O_2_. Selective media, promoting the growth of specific bacteria, may also allow for growth of other bacteria (Holck et al. 2014). Possibly, lactic acid bacteria can grow on the selective media for *Brochothrix* sp. The analysis included 10 randomly selected colonies per sample, and results should be interpreted accordingly.

#### 3.2.5 Odor Assessment

**FIGURE 9:**
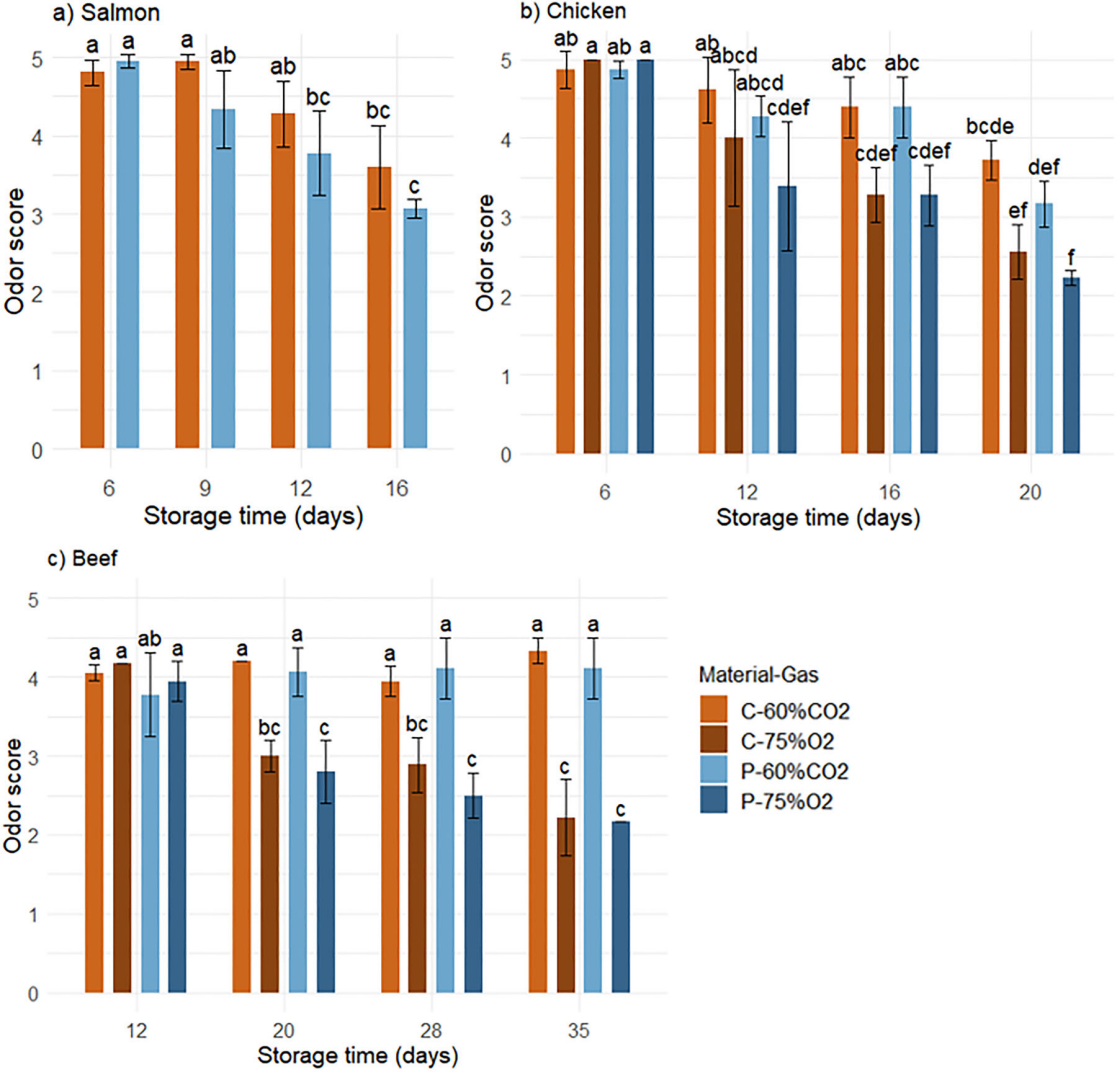
Odor score of raw salmon (a), chicken (b), and beef (c) after 6–35 days of storage under 4°C/78% RH, packaged in cellulose/PE/EVOH (“C”) and PET (“P”) trays with 60% CO_2_/40% N_2_ (“60%CO2”) and 75% O_2_/25% CO_2_ (“75%O2”). A score of 5 describes a fresh odor, 3 was the acceptability threshold, and 1 describes spoiled odor (unacceptable for consumption). Significant differences between samples are indicated by letters (those sharing the same letter are not significantly different with *p* < 0.05). Values are given as means with standard deviation represented by the error bars.

The odor score of all products decreased during storage (Figure 9). The microbial spoilage potential and the production of off‐odors depend on the type of bacteria present. Lactic acid bacteria can give sour/acidic off‐odors. The dominating bacteria in all packaging solutions, *Carnobacterium* sp., can contribute to sensory spoilage of modified‐atmosphere‐packaged meat and fish products, depending on the species and strains (Hansen et al. 2021; Laursen et al. 2006; Leisner et al. 2007). *Carnobacterium* sp. has been reported to lead to high levels of fermented/sour, pungent, and cloying off‐odors on raw chicken fillets in 60% CO_2_ (Hansen et al. 2023). Beef inoculated with *Carnobacterium maltaromaticum* showed a wide range of volatile compounds during chilled storage, likely contributing to meat spoilage (Ercolini et al. 2009).

The overall ANOVA showed a main effect of material on the odor score of salmon. However, there were no significant differences between materials at the same sampling time (according to one‐way ANOVA). Possibly, the effect of material is linked to the gas barrier properties of the materials. The higher OTR of PET compared to cellulose trays may have increased the availability of oxygen to take part in oxidation reactions or influence microbial activity, potentially leading to off‐odors like rancidity (Sivertsvik et al. 2002). The relatively high content of unsaturated fat in salmon makes it susceptible to lipid oxidation, which can form peroxides leading to off‐odors (McMillin 2008).

The odor of chicken and beef was significantly affected by gas composition. Additionally, the interaction between gas and time was significant for beef. The reduction in odor score was more prominent in 75% O_2_ compared to 60% CO_2_. This was likely related to the effect of gas composition on microbial growth and activity, as well as oxidation reactions. The headspace atmosphere can influence the spoilage potential of specific bacteria, as CO_2_ can inhibit their production of off‐odors, according to Hansen et al. (2023). The higher levels of *Brochothrix* sp. in 75% O_2_ compared to 60% CO_2_ may have contributed, as these bacteria are well‐known to cause meat spoilage. The activity of *Brochothrix thermosphacta* depends on the headspace gas, as the production of acetoin‐diacetyl, with a spoilage‐related offensive odor, increases with increasing oxygen concentration, while under anaerobic conditions, mainly lactic acid is produced (Pin et al. 2002). Sarfraz et al. (2021) found that chicken packaged in 75% O_2_ showed higher intensities of the negative odor attributes *cloying* and *fermented*, compared to chicken in 60% CO_2_ gas.

The availability of oxygen in 75%O_2_ packages can also lead to oxidation reactions forming undesired volatiles and off‐odor in meat and fish. The sensory quality of meat is largely associated with lipid oxidation. Lipid oxidation susceptibility is correlated to the degree of unsaturated fatty acids, which is higher in poultry compared to other meat products (Domínguez et al. 2019). However, the effect of gas composition on odor was more pronounced in beef compared to chicken. For beef in 75% O_2_, the odor acceptability threshold (score 3) was passed after 20 days of storage, while those in 60% CO_2_ were still rated >4 after 35 days of storage. For beef packaged in high‐oxygen atmospheres, oxidation reactions can lead to deterioration of beef flavor, as the intensity of abnormal and rancid flavors increases during storage (Campo et al. 2006). High oxygen atmosphere is commonly used to obtain the fresh red color of beef, through oxidation of myoglobin. Previous studies have reported a link between lipid oxidation and myoglobin oxidation (Murphy et al. 2013).

Material selection is essential for the packaging's functionality to protect and maintain food quality, but sustainable packaging should also include other relevant aspects. These involve consumer acceptance and willingness to separate the plastic from the cellulose part for proper recycling. Currently, multilayer materials typically used for fresh meat packaging by MAP can be challenging to recycle (Kaiser et al. 2018). The mono‐PET material included in the current study is suitable for mechanical recycling. A Life Cycle Assessment (LCA) could be performed to quantify the actual CO_2_ emissions associated with cellulose‐based trays compared to conventional plastic packaging for fresh meat.

While the PE/EVOH coating was expected to prevent moisture and fat transfer from the food into the cellulose fiber network, the substantial reduction in mechanical properties for the different foods suggested otherwise. Fat content appeared to play an important role in food–packaging interactions that influenced functional material properties. Future work could evaluate barrier integrity and suitability of such materials in contact with other food products. Furthermore, evaluation beyond the tested storage periods could determine whether the impact of food contact increases over time.

This study provides new insights into the practical suitability of cellulose‐based trays. Nevertheless, certain limitations of the study should be acknowledged. While results suggest that salmon is more challenging in terms of functional material properties (compared to chicken and beef), it should be further explored whether (and if so, how) this is attributed to the fat content. Due to the complexity of foods, isolating single food characteristics can be difficult in practical studies using real foods. In addition to the PE/EVOH coating, mainly based on a polyolefin (PE), other coatings could have been studied, such as PET. Furthermore, the bacterial counts (TVC) increased relatively fast during storage, complicating general conclusions.

## 4 Conclusion

This study evaluated the suitability of cellulose‐based trays for MAP and refrigerated storage of fresh muscle foods. The trays underwent significant changes in appearance and mechanical properties during storage, particularly when packaged with salmon. While RH is a known factor, our findings highlight that the type of product also critically influences the mechanical stability and compression strength of cellulose‐based packaging. Poor adhesion between the PE/EVOH coating and cellulose substrate led to air pocket formation in most trays during storage.

Despite these structural changes, the cellulose‐based trays maintained food quality (salmon, chicken, and beef) comparably to PET trays. This suggests that while the trays may deform during storage—posing potential challenges for logistics and consumer handling—their performance in preserving food freshness remains uncompromised. Thus, they could serve as a sustainable alternative to conventional plastics, provided that improvements in the material's durability are addressed.

Cellulose‐based trays with plastic coatings demonstrate the potential of reducing plastic use while preserving food quality and enabling recyclability. Future research should focus on enhancing their mechanical stability to minimize deformation, assessing the environmental impact of these hybrid materials through life cycle assessments, as well as evaluating consumer acceptance of these partly bio‐based and recyclable packaging alternatives.

## Author Contributions

**Agnete Jordhøy Lindstad**: conceptualization, investigation, writing – original draft, methodology, visualization, writing – review and editing, formal analysis, data curation. **Kloce Dongfang Li**: methodology, investigation, writing – review and editing. **Nusrat Sharmin**: methodology, writing – review and editing. **Marit Kvalvåg Pettersen**: methodology, conceptualization, investigation, writing – original draft, writing – review and editing, formal analysis, supervision.

## Conflicts of Interest

The authors declare no conflicts of interest.

## Supporting information




**Supplementary Figure**: jfds70505‐sup‐0001‐SuppMat.docx
