# Supplementary material for: Suitability of Cellulose‐Based Trays With PE/EVOH Coating for Modified Atmosphere Packaging of Salmon, Chicken, and Beef
Source: J Food Sci. 2025 Sep 1;90(9):e70505. doi: 10.1111/1750-3841.70505 (PMC12400135; doi:10.1111/1750-3841.70505)
Supplement: Supplementary file 1 — Supplementary Figure: jfds70505‐sup‐0001‐SuppMat.docx [file JFDS-90-0-s001.docx]

**Suitability of cellulose-based trays with PE/EVOH coating for modified atmosphere packaging of salmon, chicken, and beef**

Agnete Jordhøy Lindstad*, Kloce Dongfang Li, Nusrat Sharmin, Marit Kvalvåg Pettersen

**Supplementary materials**

Figure S.1: The compression curves (load versus time) of cellulose (“C”) and PET (“P”) trays before (“reference”) and after 16, 20 and 35 days of storage with 60%CO_2_/40%N_2_ under 4°C/78%RH with salmon, chicken and beef, respectively. One representative sample is presented for each variety.
